# Effect of Functional Electrical Stimulation of the Gluteus Medius during Gait in Patients following a Stroke

**DOI:** 10.1155/2020/8659845

**Published:** 2020-11-19

**Authors:** Sota Araki, Masayuki Kawada, Takasuke Miyazaki, Yuki Nakai, Yasufumi Takeshita, Yuta Matsuzawa, Yuya Yamaguchi, Akihiko Ohwatashi, Ryuji Tojo, Toshihiro Nakamura, Shintaro Nakatsuji, Ryoji Kiyama

**Affiliations:** ^1^Doctoral Program, Graduate School of Health Sciences, Kagoshima University, 8-35-1 Sakuragaoka, Kagoshima City, Kagoshima 890-8544, Japan; ^2^Department of Rehabilitation, Acras Central Hospital, 1-121-5 Takeoka, Kagoshima City, Kagoshima 890-0031, Japan; ^3^Course of Physical Therapy, School of Health Sciences, Faculty of Medicine, Kagoshima University, 8-35-1 Sakuragaoka, Kagoshima City, Kagoshima 890-8544, Japan; ^4^Master's Program, Graduate School of Health Sciences, Kagoshima University, 8-35-1 Sakuragaoka, Kagoshima City, Kagoshima 890-8544, Japan

## Abstract

Many stroke patients rely on cane or ankle-foot orthosis during gait rehabilitation. The purpose of this study was to investigate the immediate effect of functional electrical stimulation (FES) to the gluteus medius (GMed) and tibialis anterior (TA) on gait performance in stroke patients, including those who needed assistive devices. Fourteen stroke patients were enrolled in this study (mean poststroke duration: 194.9 ± 189.6 d; mean age: 72.8 ± 10.7 y). Participants walked 14 m at a comfortable velocity with and without FES to the GMed and TA. After an adaptation period, lower-limb motion was measured using magnetic inertial measurement units attached to the pelvis and the lower limb of the affected side. Motion range of angle of the affected thigh and shank segments in the sagittal plane, motion range of the affected hip and knee extension-flexion angle, step time, and stride time were calculated from inertial measurement units during the middle ten walking strides. Gait velocity, cadence, and stride length were also calculated. These gait indicators, both with and without FES, were compared. Gait velocity was significantly faster with FES (*p* = 0.035). Similarly, stride length and motion range of the shank of the affected side were significantly greater with FES (stride length: *p* = 0.018; motion range of the shank: *p* = 0.026). Meanwhile, cadence showed no significant difference (*p* = 0.238) in gait with or without FES. Similarly, range of motion of the affected hip joint, knee joint, and thigh did not differ significantly depending on FES condition (*p* = 0.115‐0.529). FES to the GMed and TA during gait produced an improvement in gait velocity, stride length, and motion range of the shank. Our results will allow therapists to use FES on stroke patients with varying conditions.

## 1. Introduction

Strokes can cause impairments in gait kinematics, such as a drop foot, decreased knee flexion during the swing phase, lateral trunk fluctuation, impaired ability to shift weight, and reduced leg extension angle during the stance phase [1–5]. Drop foot and decreased knee flexion are associated with decreased foot clearance [6], and reduced leg extension angle relates to short stride length and decreased propulsion force at late stance [7, 8]. These impairments impact walking ability (for example, by reducing walking speed), leading to falls and reduced walking endurance [9, 10]. Slow gait speed is reported to shorten the predicted life span and limit the spatial extent of mobility in daily life [11, 12]. Therefore, interventions to improve impaired kinematics and gait speed are important during rehabilitation in poststroke patients.

Neurorehabilitation tools, such as functional electrical stimulation (FES) [13], transcranial magnetic stimulation [14], and robot-assisted rehabilitation [15], are now being used. Among them, FES during gait is widely used in clinical practice due to its cost effectiveness and ease of use [16]. FES to the lower limb during gait training is mainly used to elicit activation of the tibialis anterior (TA) of the affected side to prevent drop foot during the swing phase, and to correct active walking. Previous studies regarding FES during gait report that it improves spatiotemporal aspects and kinematics of gait movement; it also increases motor evoked potential and cortical input [17, 18]. Taken together, FES to the TA may potentially influence not only peripheral but also corticomotor plasticity.

Lateral trunk fluctuation and difficulties in weight shift to the affected side during the stance phase are closely related in stroke patients, and are caused mainly by impaired lateral stability of the affected hip joint [3, 19, 20]. The hip abductor mainly controls the lateral stability of the hip joint; it generates the medial ground reaction for balancing the centre of mass on the support base during gait [21, 22]. Therefore, FES to the gluteus medius (GMed) as well as the TA would increase lateral stability of the affected hip joint during gait, resulting in an increase in stride length and gait velocity. However, FES applied to the GMed and TA has been investigated in only a few studies, and its effects on gait kinematics are unclear [23–25].

Other assistive walking devices, including a cane and ankle-foot orthosis (AFO) improve kinematics of the affected lower limb and stability during rehabilitative walking in poststroke patients. Canes contribute to an improvement in gait speed, step length, and symmetry [26, 27], and an AFO immediately improves drop foot during the swing phase [28, 29]. Therapists recommend the use of an AFO and a cane for stroke patients during gait exercises. Thus, the combined use of FES to the GMed and TA with an assistive walking device may facilitate the learning of an effective gait pattern during gait rehabilitation in patients following a stroke.

However, previous studies that have investigated the effect of FES on gait performance have only analysed participants who were able to walk without assistive devices. To our knowledge, there is no literature pertaining to the effect of gait training in stroke patients including those using assistive devices. The purpose of this study was to investigate the immediate effect of FES on the GMed and TA in stroke patients, including those who relied on assistive devices to walk. The hypothesis of this study was that FES to the GMed and TA improves stability during the stance phase of the affected lower limb and increases hip extension angle, stride length, and gait speed. Combined use of FES and assistive walking device would create a wider intervention for gait training in stroke patients under various conditions.

## 2. Materials and Methods

### 2.1. Participants

The recruitment of participants and data collection were conducted from October 2018 to September 2019. Fourteen stroke patients were enrolled in this study, and all of them were right-handed (7 males; mean poststroke duration: 194.9 ± 189.6 d; mean age: 72.8 ± 10.7 y; mean Fugl-Meyer's assessment of lower extremity: 25.9 ± 6.0; Table 1). All patients have been receiving standard physical therapy (e.g., facilitation exercises and balance and gait training) for 1 hour/day at more than 3 times a week from the onset of stroke. All participants except one used a cane, and half of the participants used an AFO and a cane simultaneously. Participants were inpatients or outpatients receiving physical therapy and occupational therapy in Acras Central Hospital, Kagoshima, Japan. The sample size was calculated based on the effect size obtained from a previous report using G∗Power 3.1.9.2 [30]. The power analysis indicated that at least 13 participants were required to achieve a power of 0.80 at *p* < 0.05. The inclusion criteria were as follows: (1) first onset of poststroke hemiparesis, (2) the ability to walk at least 14 m without assistance, and (3) stable medical condition. The exclusion criteria were as follows: (1) severe cardiopulmonary disease and (2) severe sensory disturbance, severe ataxia, or severe higher brain dysfunction. Prior to the investigations, all patients provided written informed consent for participation in the study. This study was approved by the Ethics Committee of Acras Central Hospital (no. 0008). The study was registered with the University Hospital Medical Information Network Clinical Trial Registry (UMIN-CTR000034580).

### 2.2. Measurement and Procedures

Speed and motion of the lower limbs during gait with and without FES to the GMed and TA after an adaptation period were compared. The motion of the lower limb was measured using magnetic inertial measurement units (IMU; MTw Awinda, Xsens, Enschede, NL) attached by elastic belts to the posterior pelvis, the anterior thigh of the affected side, and both anterior shanks. IMU consist of a 3D rate gyroscope, a 3D accelerometer, and a 3D magnetometer; they calculate the Euler angles with a sampling frequency of 100 Hz. IMU attached to the bilateral shanks were also used to identify walking events.

**Figure 1:**
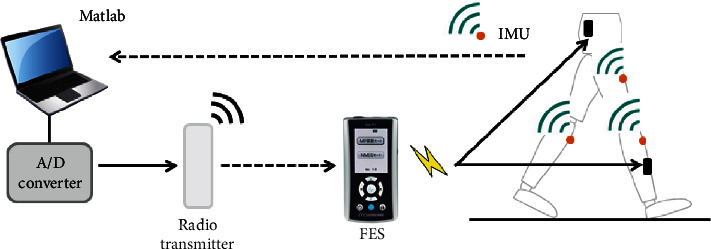
Schematic diagram of functional electrical stimulation (FES) control. The solid line represents wired processing, and the broken line represents wireless processing. The FES was held by the patient. Orange dots mark the position of the magnetic inertial measurement units (IMU).

A surface FES system (NM-F1, Ito Co., Ltd., Saitama, Japan), where stimulation timing could be controlled by a radio transmitter, was used in this study. Frequency and pulse width were 40 Hz and 200 *μ*s, respectively [23, 24]. One of the electrodes for GMed was placed over the line between the posterior superior iliac spine and the greater trochanter, and the other electrode was placed over the line connecting the highest point of the iliac crest and the greater trochanter. One of the electrodes for TA was positioned just distal and anterior to the head of the fibula, and the other electrode was positioned over the tibialis anterior muscle belly. Stimulation timing was controlled based on initial contact (IC) of both legs as detected dependent on the Euler angle as measured by IMU from attachments to bilateral shanks. Validity of the detection of initial contact from the shank tilt angle was tested in a previous study [31, 32]. Shank tilt angle measured by IMU was uploaded to a laptop PC in real time via Zigbee. Immediately, a custom program written by MATLAB R2019a (MathWorks Inc., MA, USA) identified the timing at which the shank tilt angle overcame the threshold and issued a signal to stimulate the muscle via an A/D converter and radio transmitter (Figure 1) [31]. The affected GMed was stimulated from the affected IC to 40% of the step time of the affected side, and the affected TA was stimulated from IC of the nonaffected side to 10% of the step time of the affected side [33]. The electrodes were attached similarly to gait with FES condition, but no electrical stimulation was applied during gait without FES.

This study used a randomized cross-over design. Participants walked 14 m at a comfortable velocity with and without stimulation to the GMed and TA. Each measurement was performed on separate days and at least one day apart (average 4.9 ± 5.0 d). Walk tests under two conditions were conducted at random using a random number list. These tests, with and without FES, were performed after an adaptation period to achieve a familiar gait condition. During the adaptation period, participants walked under similar conditions to those in the walk test at the rehabilitation centre for 10 or 20 minutes. The duration of walking adaptation was determined so that participants could become familiar with the FES and without inducing fatigue as suggested in previous studies [25].

Motion range of tilt angle of the affected thigh and shank segments in the sagittal plane, motion range of the affected hip and knee extension-flexion angle, step time, and stride time were all calculated from IMU data during the middle ten walking strides. Time taken to walk the middle 10 m was also recorded using a stopwatch. Walking speed, cadence, and stride length were calculated. Data analysis was performed using MATLAB [34].

### 2.3. Statistical Analysis

We compared the gait velocity, cadence, stride length, motion range of the thigh and shank tilt angle, and motion range of the hip and knee joint angle during walking with and without FES. The Shapiro-Wilk test was used to test for the normality of distribution of all data. Subsequently, gait parameters were tested by a paired *t*-test when normality could be assumed. When normality could not be assumed, the Wilcoxon signed-rank test was used for comparison between two conditions. Statistical analyses were performed using SPSS 25.0 for Windows (IBM, NY, USA), and the significance was set at *p* < 0.05.

## 3. Results

**Figure 2:**
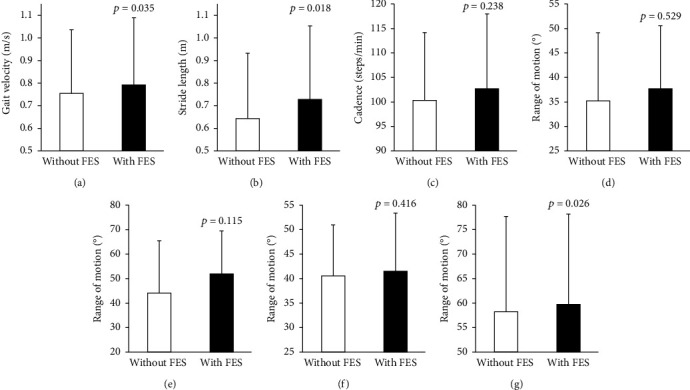
Average and standard deviation bars for gait parameters without and with FES. (a) Gait velocity. (b) Stride length. (c) Cadence. (d) Range of motion of affected hip. (e) Range of motion of affected hip knee. (f) Range of motion of affected thigh. (g) Range of motion of affected shank.

Gait velocities were 0.755 ± 0.281 m/s and 0.794 ± 0.295 m/s in gait without and with FES, respectively, showing a significantly faster velocity in gait with FES (Figure 2(a), *p* = 0.035). Similarly, stride length was 0.644 ± 0.287 m and 0.723 ± 0.329 m (Figure 2(b)), and motion range of the shank of the affected side was 58.1 ± 19.5° and 59.8 ± 18.3° (Figure 2(g)) in gait without and with FES, respectively, indicating significantly greater values in gait with FES (stride length: *p* = 0.018; motion range of the shank: *p* = 0.026).

Cadence was 100.1 ± 13.9 steps/minute and 102.5 ± 15.1 (Figure 2(c)) in gait without and with FES, showing no significant difference (*p* = 0.238). Similarly, range of motion of the affected hip joint, knee joint, and thigh did not differ significantly depending on FES condition (Figures 2(d)–2(f), *p* = 0.115‐0.529).

## 4. Discussion

This study verified the immediate effects of FES to the GMed and TA on gait performance in stroke patients, including those who needed assistive devices. The present results showed that FES to the GMed and TA during gait increased gait speed, stride length, and range of motion of the affected shank, and therefore supported our hypothesis. Meanwhile, there was no significant difference in hip and knee joints dependent on FES; this finding was inconsistent with our expectations.

To date, studies of FES during gait usually stimulate only the TA in stroke patients who could walk without an assistive device, and these reports show that FES increases gait speed, stride length, and cadence compared to spontaneous gait [35, 36]. Our results also agreed with these studies, and additional stimulation to the GMed emphasised the immediate effect of FES on walking performance. Decreased muscle strength in an affected lower limb is the main impairment in stroke patients [37]. The GMed contributes to the control of lateral stability during the stance phase, and low GMed strength is associated with decreased gait speed [38]. Electrical stimulation to the GMed and TA during gait would increase stability during the paretic stance phase and decrease any difficulty in forward displacement of the affected lower limb, leading to an increase in range of motion of the affected shank and stride length. Moreover, the increase of leg extension angle including hip extension correlates with propulsive force [8]. Thus, an increase in stride length as a result of FES improved gait speed in our study.

Furthermore, our results implicated the synergetic effects of FES and gait-assistive devices on gait performance in stroke patients. In the present study, all but one of the participants used a cane, and half of the participants used an AFO and a cane simultaneously. The use of assistive devices contributes to improved clearance in the swing phase and increases symmetry and stability of gait [39]. Improved stability in the stance phase depended on FES to the GMed and TA; utilization of an AFO and a cane would enable stroke patients to select a strategy of increasing stride length rather than increasing cadence to increase gait speed. Therapists usually recommend patients who have had a stroke to use assistive devices during gait rehabilitation. Most studies to date have explored the effect of FES on gait in stroke patients without a cane and AFO conditions [23–25]; therefore, the synergetic effect of FES and a gait-assistive device on gait performance has not previously been investigated. Our results regarding the synergetic effect of FES and a gait-assistive device will allow therapists to use FES on stroke patients with varying conditions.

Our study showed that an increased range of motion of the affected shank could be achieved during gait, but not in other joint angles. One possible explanation for this is that strategies for extending stride length varied depending on each individual. Stride length depends on the motion in the sagittal plane of the hip, knee, and ankle joint of affected and unaffected lower extremities of stroke patients. The present study could not clarify the factor of increase in stride length due to a lack of analysis of motion on unaffected lower extremities.

Meanwhile, the current study had some limitations. This study verified the immediate effect of FES on the GMed and TA on gait performance in stroke patients, but the effect of long-term intervention was not analysed. Heterogeneity in the use of gait-assistive devices of participants made it difficult to distinguish the effects of FES, AFO, and canes. Therefore, further studies, including a more detailed motion analysis of lower extremities for a larger stroke population, with adjustment for poststroke duration, under various gait conditions are needed. Such studies should clarify the synergetic effects of FES on the GMed and TA and gait-assistive devices on gait performance in stroke patients.

## 5. Conclusions

This study examined the effects of FES on the GMed and TA during gait in stroke patients, including those who relied on assistive devices to walk. The results showed an improvement in gait speed, stride length, and shank range of motion, implicating the synergetic effects of FES and gait-assistive devices on gait performance. This information will allow therapists to use FES on stroke patients with varying conditions.

## Figures and Tables

**Table 1:** Characteristics of stroke patients.

No.	Age (y)	Sex	Affected side	Poststroke duration (d)	FMA	Use of AFO	Use of cane	Gait adaptation period (min)
1	84	F	R	200	31	Yes	Yes	10
2	77	F	R	98	32	No	Yes	10
3	78	M	L	71	34	No	Yes	20
4	84	M	R	151	17	Yes	Yes	10
5	74	M	R	276	16	Yes	Yes	20
6	79	M	R	141	23	Yes	Yes	10
7	89	M	R	68	24	No	Yes	20
8	77	F	L	131	29	No	Yes	20
9	49	F	R	58	28	No	Yes	10
10	62	F	R	794	15	Yes	Yes	10
11	73	F	R	322	24	Yes	Yes	10
12	67	F	R	54	30	No	Yes	20
13	69	M	L	59	30	No	No	20
14	57	M	R	57	29	Yes	Yes	20

F: female; M: male; R: right; L: left; FMA: Fugl-Meyer's assessment of lower extremity; AFO: ankle-foot orthosis.

## Data Availability

The data used to support the findings of the current study are available from the corresponding author upon request.
